# 115. A New Strategy Against Healthcare-Associated Infections: A Tertiary Academic Healthcare System-wide Surveillance of Hospital-Onset Bacteremia and Fungemia

**DOI:** 10.1093/ofid/ofaf695.047

**Published:** 2026-01-11

**Authors:** Sean H P Jung, Aryeh Feldheim, Shira Abeles, Frank Myers, Francesca J Torriani

**Affiliations:** VA San Diego / UC San Diego, San Diego, CA; UC San Diego, San Diego, California; University of California, San Diego, San Diego, CA; UC San Diego, San Diego, California; University of California, San Diego School of Medicine, San Diego, California

## Abstract

**Background:**

Healthcare-associated infections (HAIs) are recognized as a substantial cause of preventable harm and are associated with excess morbidity and mortality in patients. National Healthcare Safety Network(NHSN) has proposed surveillance of Hospital-onset Bacteremia and Fungemia (HOB) to increase the yield of bloodstream infections (BSI) and care improvement opportunities in acute care hospitals. We sought to quantify and investigate the causes of HOBs at a large academic acute care hospital system.

**Table 1.**
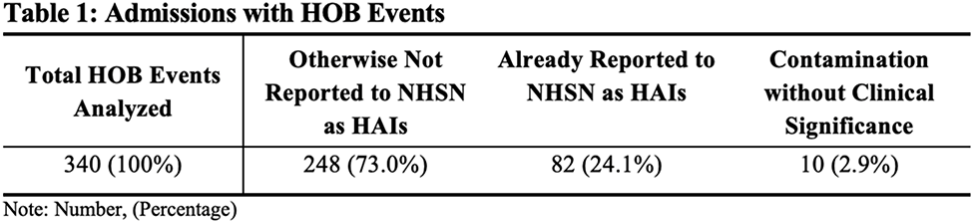
Admissions with HOB Events Note: Number, (Percentage)

**Methods:**

HOB events, defined as bacterial or fungal pathogens isolated from a blood culture collected between 7/1/23 and 6/30/24 on the 4th calendar day of admission (HD4) or later, were retrospectively reviewed. Only one event was recorded per admission. The NHSN laboratory-confirmed bloodstream infection (LCBI)-2 definition was used to distinguish contamination from infection due to common commensals.

**Table 2.**
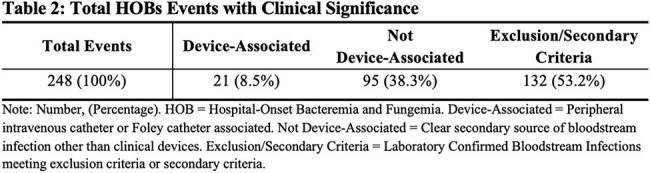
Total HOBs Events with Clinical Significance Note: Number, (Percentage). HOB = Hospital-Onset Bacteremia and Fungemia. Device-Associated = Peripheral intravenous catheter or Foley catheter associated. Not Device-Associated = Clear secondary source of bloodstream infection other than clinical devices. Exclusion/Secondary Criteria = Laboratory Confirmed Bloodstream Infections meeting exclusion criteria or secondary criteria.

**Results:**

A total of 381 bacteremia events (BE) were identified. Of those, 41 BE were presented on admission and were excluded, leaving 340 potential HOB events (Figure 1). Eighty-two were reported to NHSN as HAIs, and ten were deemed contamination without clinical significance. Two hundred forty-eight HOBs did not meet NHSN HAI criteria for reporting (Table 1). Of those, 132 met LCBI definitions but met exclusion criteria or secondary criteria. The remaining 116 cases were further evaluated for potentially preventable or non-preventable causes of BSIs. Twenty-one cases were deemed device-associated such as peripheral intravenous catheters or Foley catheters however not matching CAUTI criteria. Ninety-five cases were not device-associated (Table 2).

**Conclusion:**

HOB surveillance has identified a significant portion of clinically relevant device-associated bloodstream infections, such as those related to peripheral intravenous (PIV) lines, that are currently excluded from the NHSN HAI surveillance system. The nationwide implementation of a simplified measure for healthcare-associated bloodstream infections may help healthcare systems concentrate on more effective quality improvement initiatives.

**Disclosures:**

All Authors: No reported disclosures

